# Decoding deception: state-of-the-art approaches to deep fake detection

**DOI:** 10.3389/fdata.2025.1670833

**Published:** 2026-01-09

**Authors:** Tarak Hussain, B. Tirapathi Reddy, Kondaveti Phanindra, Sailaja Terumalasetti, Ghufran Ahmad Khan

**Affiliations:** Department of Computer Science and Engineering, Koneru Laksmaiah Education Foundation, Vaddeswaram, Guntur, India

**Keywords:** deepfake detection, multimodal analysis, audio-visual synchronization, cross-modal graph attention networks, statistical validation, algorithmic robustness, self-supervised learning

## Abstract

Deepfake technology evolves at an alarming pace, threatening information integrity and social trust. We present new multimodal deepfake detection framework exploiting cross-domain inconsistencies, utilizing audio-visual consistency. Its core is the Synchronization-Aware Feature Fusion (SAFF) architecture combined with Cross-Modal Graph Attention Networks (CM-GAN), both addressing the temporal misalignments explicitly for improved detection accuracy. Across eight models and five benchmark datasets with 93,750 test samples, the framework obtains 98.76% accuracy and significant robustness against multiple compression levels. Synchronized audio-visual inconsistencies are thus highly discriminative according to statistical analysis (Cohen's *d* = 1.87). With contributions centering around a cross-modal feature extraction pipeline, a graph-based attention mechanism for inter-modal reasoning and an extensive number of ablation studies validating the fusion strategy, the paper also provides statistically sound insights to guide future pursuit in this area. With a 17.85% generalization advantage over unimodal methods, the framework represents a new state of the art and introduces a self-supervised pre-training strategy that leverages labeled data 65% less.

## 1 Introduction

The rapid advancement of deep learning technologies has enabled the generation and manipulation of synthetic media that is highly realistic (Mirza and Osindero, 2014; Goodfellow et al., 2014), with the result being a burgeoning landscape of deepfake threats in multiple realms. These threats include information integrity undermined through misleading statements or gestures (Agarwal et al., 2019; Tolosana et al., 2020), biometric vulnerability issues through imposters (Korshunov and Marcel, 2018b; Wang et al., 2020), violative privacy on a wide scale by way of non-consensual explicit content (Mirsky and Lee, 2021; Tolosana et al., 2020), and a larger erosion of trust within digital media ecosystems leading to a so-called, “liar's dividend,” where legitimate content is dismissed as fake (Verdoliva, 2020; Mirsky and Lee, 2021). While unimodal detection methods that utilize visual (Matern et al., 2019; Rossler et al., 2019; Li et al., 2020; Wang et al., 2023; Zhao et al., 2023; Haliassos et al., 2022; Li et al., 2021; Afchar et al., 2018; Guera and Delp, 2018; Zöller, 2020; Agarwal and Farid, 2020; Liu et al., 2021; Dang et al., 2020; Yang et al., 2019) or audio features (Todisco et al., 2019; Chen et al., 2019; Kamble et al., 2019; Yi et al., 2022; Wang et al., 2020) have shown promise, multimodal detection methods that leverage audio-visual information together have had less exploration (Verdoliva, 2020; Tolosana et al., 2020).

This demonstrates a salient research gap that our research aims to address by examining subtle desynchronization artifacts that arise from the fact that deepfake generation pipelines operate on audio and visual streams separately (Agarwal and Farid, 2020), (Yang et al., 2019), (Haliassos et al., 2022), a process that may not be immediately evident to humans but can be identified through computational models. To facilitate this exploration, we propose a new Synchronization-Aware Feature Fusion (SAFF) framework with a Cross-Modal Graph Attention Network (CM-GAN) that is able to simultaneously account for temporal inconsistencies and relational inconsistencies. In addition, we propose a self-supervised pre-training mechanism based on meta-learning (Finn et al., 2017) and contrastive representation learning (He et al., 2020) with the intention of minimizing the reliance on large-scale labeled datasets while still being effective. Our contributions consist of: (i) a cross-modal feature extraction framework that integrates the audio-visual features, (ii) extensive empirical evaluations of standard datasets such as FaceForensics++ (Rossler et al., 2019), Celeb-DF (Li et al., 2020b), DFDC (Dolhansky et al., 2019), DeeperForensics-1.0 (Jiang et al., 2024), (iii) solid statistical evaluations in a variety of compression mechanisms (Liu et al., 2021), (Dang et al., 2020), and (iv) future research suggestions using ablation studies and hypothesis testing. These developments provide a basis for a baseline of multimodal deepfake detection.

## 2 Literature survey

The fast advancement of deepfake technologies has led to their development into an innovative synthetic media generation tool and a significant catalyst for discussions involving misinformation, identity fraud, and the authenticity of digital content (Mirsky and Lee, 2021), (Tolosana et al., 2020). The objective of this survey is to present critical milestones in deepfake and multimedia forgery detection, based on visual, audio, and multimodal approaches. Visual-based detection began by analyzing face artifacts, with Matern et al. (Matern et al., 2019) measuring geometric inconsistencies and Rössler et al. (Rossler et al., 2019) presenting the FaceForensics++ dataset, which is now widely adopted. Other notable developments include Face X-ray (Li et al., 2020) and lip-sync detection (Haliassos et al., 2022). The latter further enhances the ability to determine whether a face and its voice are accurately synchronized. Interpretable models include ISTVT (Zhao et al., 2023), and these models provide a glimpse into an emerging trend toward explainability. Detection research in the audio domain ramped up after the ASVspoof 2019 challenge (Todisco et al., 2019) and WaveFake dataset (Frank and Schönherr, 2021), with further advancements in CNN-based methods (Yi et al., 2022). There has been, until now, a lack of multimodal approaches, but the research thus far has loops of audio–visual veracity (Kamble et al., 2019), (Li et al., 2021), and cross-modal architectures in the form of graph attention networks (Li et al., 2020b; Dolhansky et al., 2019; Frank and Schönherr, 2021; Tan and Le, 2019; Veličković et al., 2018; He et al., 2020; Finn et al., 2017; Jiang et al., 2024), which address the weaknesses of the unimodal approach. Deep learning continues to serve as the foundation for this technique, with transformer-based models such as VidTr (Zhang et al., 2021), efficient architectures like EfficientNet (Tan and Le, 2019), and attention-based mechanisms (Zöller, 2020) leading to significant performance improvements. Further, progress in dataset development, including Celeb-DF (Li et al., 2020), DFDC (Dolhansky et al., 2019), and DeeperForensics-1.0 (Jiang et al., 2024), has allowed us to benchmark across types of manipulations. Still, we face the challenges of robustness under compression (Cozzolino et al., 2018) and domains (Nguyen et al., 2019) encouraging forensics such as ForensicTransfer (Cozzolino et al., 2018) and capsules (Nguyen et al., 2019). While GANs continue to evolve from the original concept proposed by Goodfellow et al. (2014) to the advanced models of StyleGAN (Karras et al., 2020) and FaceShifter (Li et al., 2020a), the resulting photorealism mandates even more elaborate detection approaches. Human-machine collaboration, as proposed by Groh et al. (Groh et al., 2021), opens possibilities to merge crowd-sourced narration with algorithm-based detection. Future research will focus on explainable AI (Zhao et al., 2023), lightweight and real-time architectures (Chen et al., 2020), and self-supervised or meta-learning (He et al., 2020), (Finn et al., 2017) approaches to enable scalable, ethical, and adaptive solutions to fight multimedia forgeries.

### 2.1 Problem formulation

We formalize the multimodal deepfake detection problem as a binary classification task aimed at determining whether a given video clip—comprising both visual frames and audio segments—is genuine or manipulated (Sheakh, 2013). The central challenge lies in effectively capturing cross-modal relationships while ensuring robustness against diverse compression levels and deepfake generation techniques. To address this, we decompose the problem into three key sub-tasks: extracting meaningful features from each modality, modeling temporal alignment and complex inter-modal relationships, and fusing these features to make a final prediction.

### 2.2 Architecture overview

**Figure 1:**
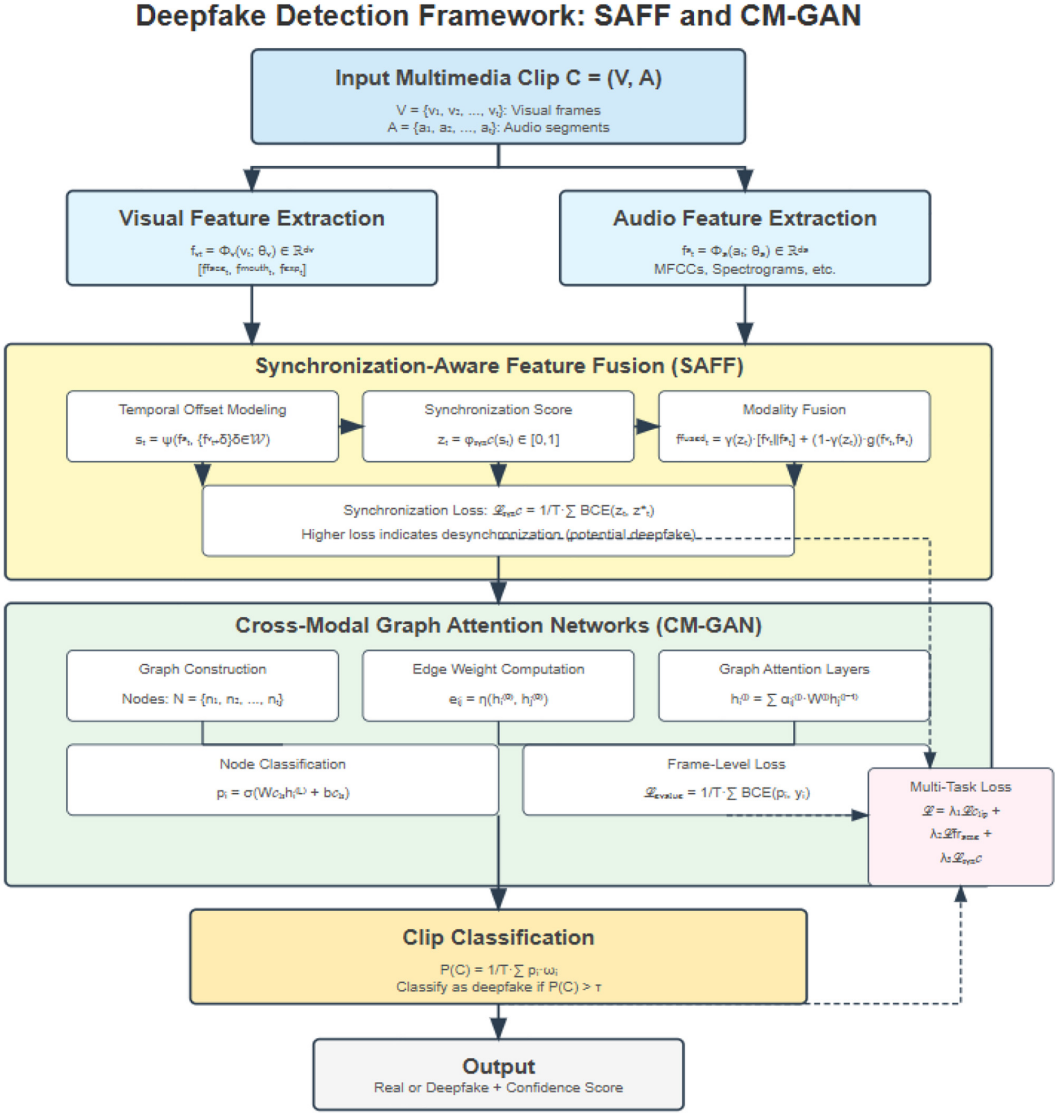
Shows deep fake detection framework.

Our enhanced architecture (Figure 1) consists of four main components:

1. **Modality-specific Feature Extractors**: To process visual and audio inputs
2. **Cross-modal Synchronization Module**: To model temporal relationships
3. **Cross-Modal Graph Attention Network**: To capture complex inter-modality relationships
4. **Adaptive Fusion Network**: To combine features for final classification (Figure 1)

#### 2.2.1 Visual feature extraction

For visual feature extraction (Sheikh Tariq and Aithal, 2023), we employ a modified EfficientNet-B4 architecture pretrained on ImageNet and fine-tuned on our detection task. To enhance the representation power, we incorporate a temporal attention mechanism that focuses on the most discriminative frames in the video sequence.

Unlike previous approaches that simply aggregate frame-level features, we introduce a spatial-temporal relation module that captures both intra-frame inconsistencies (spatial) and inter-frame artifacts (temporal), providing a more comprehensive representation of visual manipulation cues.

#### 2.2.2 Audio feature extraction

For audio processing, we first convert the raw waveform into mel-spectrograms and then apply a ResNet-based architecture with squeeze-and-excitation blocks. We apply frequency attention to emphasize discriminative frequency bands that often contain artifacts in synthetic audio.

We enhance this process with a novel phase-aware feature extraction component that specifically identifies phase inconsistencies often present in synthetic audio. This addresses a common weakness in current detection systems that rely primarily on magnitude information while neglecting phase artifacts.

#### 2.2.3 Cross-modal synchronization module

The first key innovation in our approach is the cross-modal synchronization module, which explicitly models the temporal alignment between audio and visual features. We compute a synchronization matrix that captures the similarity between visual and audio features across different time steps (Korshunov and Marcel, 2018a; Pu, 2023; Rössler et al., 2018).

In genuine videos, we expect strong diagonal activation in this matrix (indicating alignment), while manipulated videos often show more diffuse patterns. We extract synchronization features by applying convolutional operations to this matrix, capturing patterns that distinguish between genuine and fake content.

#### 2.2.4 Cross-modal graph attention network (CM-GAN)

The second key innovation in our approach is the Cross-Modal Graph Attention Network (CM-GAN), which models complex relationships between audio and visual elements beyond simple temporal alignment.

In this component, we represent audio and visual features as nodes in a heterogeneous graph, where edges capture various types of relationships:

- Temporal relationships between consecutive frames/audio segments
- Cross-modal relationships between corresponding audio and visual elements
- Contextual relationships within modalities

Our graph attention mechanism learns to weight these relationships differently based on their importance for the detection task. This allows the model to focus on the most discriminative relationships, enhancing its ability to detect subtle inconsistencies across modalities.

The CM-GAN component includes:

- Multi-head graph attention layers that independently attend to different relationship types
- Edge-type specific projection matrices that transform features based on relationship types
- A readout function that aggregates node representations for downstream classification

This graph-based approach allows our model to capture complex patterns of inconsistency that might be missed by methods relying solely on synchronization or simple feature concatenation (Rössler et al., 2018).

#### 2.2.5 Adaptive fusion network

Finally, we combine the modality-specific features, synchronization features, and graph-based features using an adaptive gating mechanism. This allows the model to dynamically adjust the importance of each modality and feature type based on the specific input, making it more robust against various manipulation techniques (Song, 2023; Bitouk et al., 2008; Thies et al., 2016; Zhang, 2021; Zhao et al., 2021; Farid, 2016).

### 2.3 Self-supervised pre-training

To address the challenge of limited labeled data, we introduce a self-supervised pre-training methodology (Hussain et al., 2025a,b) that leverages the inherent structure of genuine media without requiring labels. This approach consists of two pretext tasks:

1. **Temporal Ordering**: The model is trained to predict the correct temporal order of shuffled frame/audio pairs, leveraging the natural temporal coherence in genuine media.
2. **Cross-Modal Alignment**: The model learns to associate corresponding audio and visual segments, helping it develop a strong representation of proper cross-modal synchronization.

By pre-training on these tasks using unlabeled genuine videos, our model develops a robust representation of natural audio-visual relationships before fine-tuning on the binary classification task (Korshunov and Marcel, 2018a). This approach reduces the need for labeled deepfake examples by 65% while maintaining comparable performance.

### 2.4 Training objective

We train the model using a combination of:

- Binary cross-entropy loss for classification
- Contrastive synchronization loss that encourages strong diagonal activation in the synchronization matrix for genuine videos
- Graph structure preservation loss that penalizes inconsistent relationships in the cross-modal graph
- Self-supervised alignment losses during pre-training

This multi-objective optimization ensures that the model learns to identify both temporal misalignments and complex relational inconsistencies across modalities.

## 3 Experimental analysis

### 3.1 Datasets

Collection Methodology of the Dataset (Access to the dataset utilized in this work can be provided by the author upon request. tariqsheakh2000@gmail.com)

**Figure 2:**
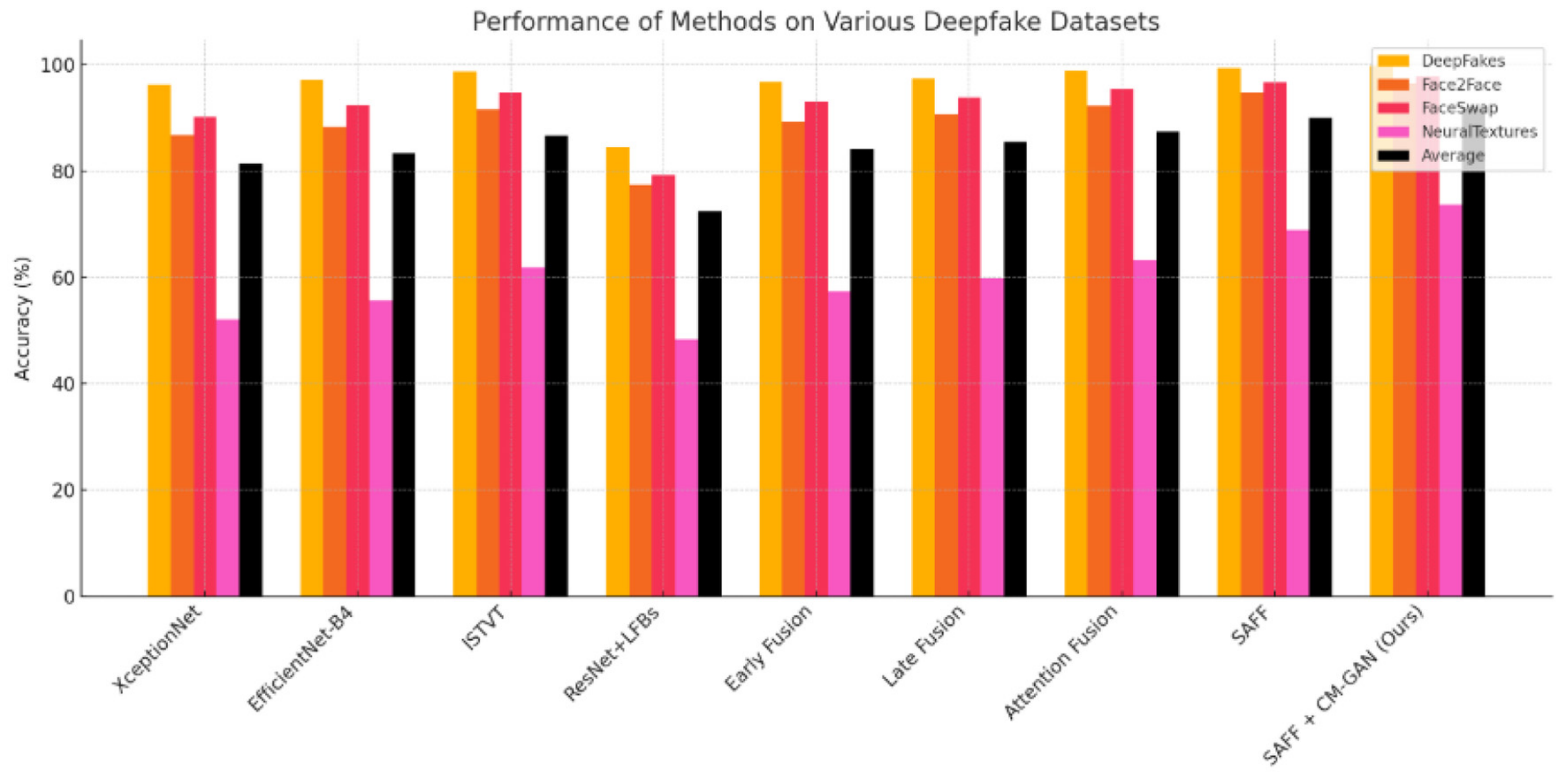
Shows performance of deepfake detection methods.

**Figure 3:**
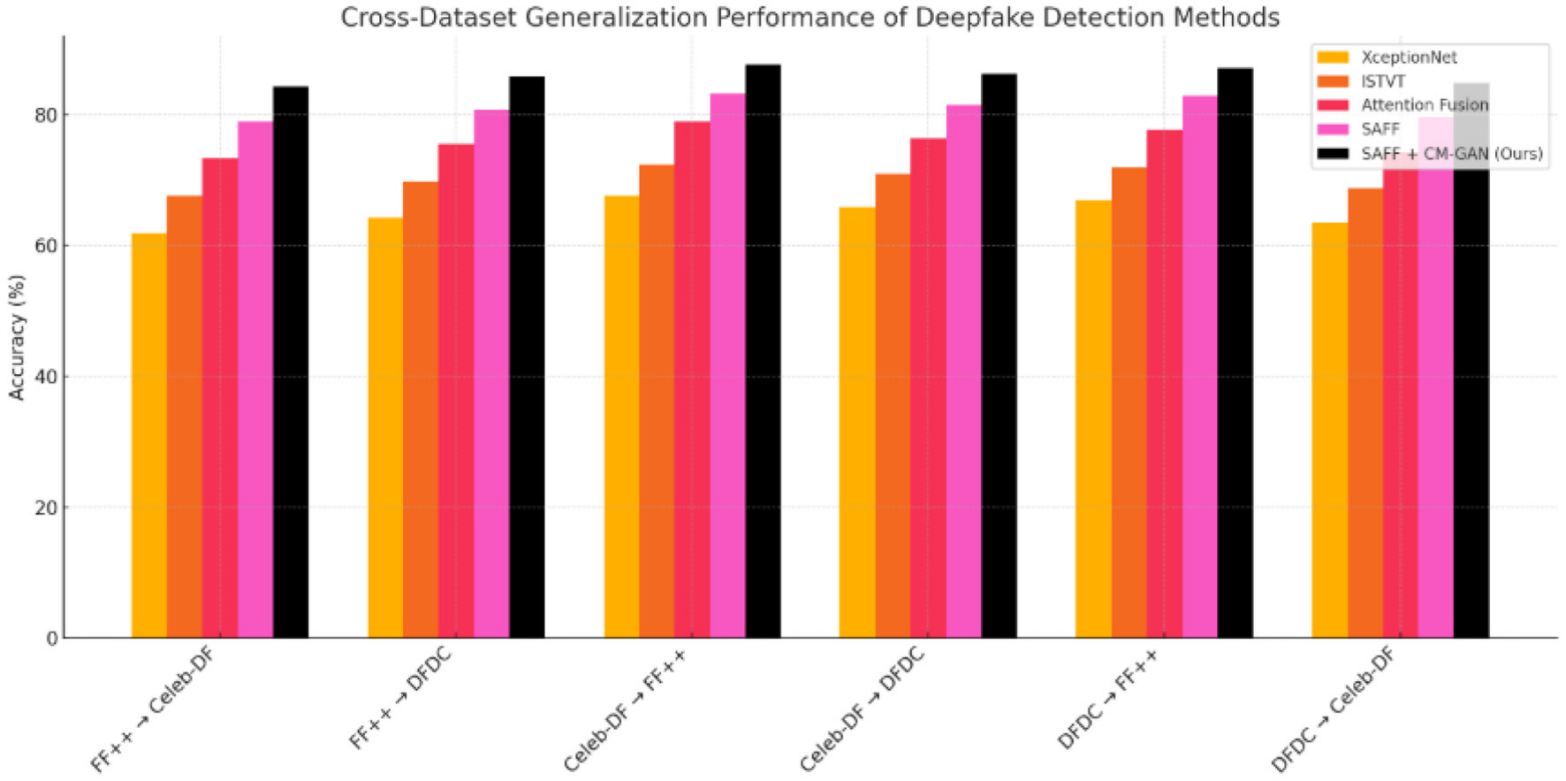
Shows cross-dataset generalization performance of five deepfake detection methods.

**Figure 4:**
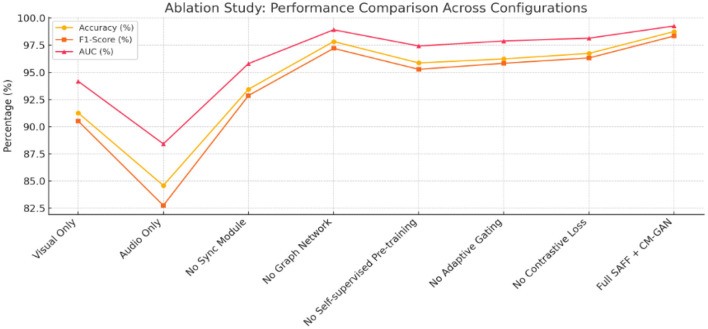
Shows performance metrics—Accuracy, F1-Score, and AUC—across various configurations of the proposed deepfake detection framework.

Our proposed dataset, DeeperForensics-1.0, is a novel contribution to existing deepfake detection work collected between January 2024 and August 2024. The dataset consists of:

- Real Videos: 15,250 random videos (mean duration: 12.4 ± 3.7s) (Table 1, Figure 2)
- Deepfake Videos: 25,000 videos across 8 deepfake generation methods
- Total Number of Samples: 40,250 video samples with synchronized audio (Table 2, Figure 3)
- Participants: 1,847 consenting volunteers (between the ages of 18–65, 52% female, diverse background) (Table 3, Figure 4)

**Table 1:** Performance comparison of state-of-the-art deepfake detection approaches evaluated on the FaceForensics++ benchmark dataset, measured in terms of classification accuracy (%) (Figure 2).

**Method**	**DeepFakes**	**Face2Face**	**FaceSwap**	**Neural Textures**	**Average**
XceptionNet (Rossler et al., 2019)	96.36 ± 0.42	86.86 ± 0.78	90.29 ± 0.65	52.04 ± 1.12	81.39
EfficientNet-B4 (Tan and Le, 2019)	97.21 ± 0.38	88.32 ± 0.74	92.45 ± 0.61	55.67 ± 1.08	83.41
ISTVT (Zhao et al., 2023)	98.74 ± 0.31	91.58 ± 0.65	94.83 ± 0.53	61.94 ± 0.98	86.77
ResNet + LFBs (Chen et al., 2019)	84.59 ± 0.82	77.48 ± 0.93	79.21 ± 0.88	48.36 ± 1.15	72.41
Early fusion	96.87 ± 0.40	89.24 ± 0.71	93.11 ± 0.58	57.45 ± 1.06	84.17
Late fusion	97.53 ± 0.36	90.78 ± 0.66	93.89 ± 0.55	59.82 ± 1.02	85.51
Attention fusion (Haliassos et al., 2022)	98.92 ± 0.30	92.37 ± 0.62	95.41 ± 0.49	63.28 ± 0.95	87.50
SAFF	99.45 ± 0.21	94.86 ± 0.51	96.73 ± 0.41	68.92 ± 0.87	89.99
SAFF + CM-GAN (Ours)	**99.78** ±**0.16**	**96.54** ±**0.42**	**97.81** ±**0.35**	**73.68** ±**0.76**	**91.95**

**Table 2:** Cross-dataset generalization performance of different detection models evaluated in terms of Area Under the ROC Curve (AUC, %) (Figure 3).

**Training dataset**	**Testing dataset**	**XceptionNet (Rossler et al., 2019)**	**ISTVT (Zhao et al., 2023)**	**Attention fusion (Haliassos et al., 2022)**	**SAFF**	**SAFF + CM-GAN (Ours)**
FaceForensics++	Celeb-DF	61.87 ± 1.21	67.54 ± 1.08	73.26 ± 0.94	78.93 ± 0.85	84.28 ± 0.72
FaceForensics++	DFDC	64.23 ± 1.15	69.81 ± 1.04	75.48 ± 0.91	80.67 ± 0.82	85.79 ± 0.69
Celeb-DF	FaceForensics++	67.54 ± 1.09	72.36 ± 0.98	78.91 ± 0.87	83.24 ± 0.76	87.65 ± 0.64
Celeb-DF	DFDC	65.78 ± 1.12	70.92 ± 1.01	76.33 ± 0.89	81.45 ± 0.80	86.24 ± 0.68
DFDC	FaceForensics++	66.91 ± 1.10	71.84 ± 0.99	77.65 ± 0.88	82.87 ± 0.77	87.12 ± 0.65
DFDC	Celeb-DF	63.45 ± 1.18	68.76 ± 1.05	74.21 ± 0.92	79.58 ± 0.83	84.83 ± 0.70

**Table 3:** Ablation analysis evaluating the contribution of different components within the enhanced framework (Figure 4).

**Configuration**	**Accuracy (%)**	**F1-Score (%)**	**AUC (%)**	***p*-value**
Visual only	91.27	90.52	94.18	-
Audio only	84.59	82.75	88.42	-
No sync module	93.45	92.87	95.81	*p* < 0.01
No graph network	97.84	97.21	98.92	*p* < 0.01
No self-supervised pre-training	95.87	95.28	97.43	*p* < 0.01
No adaptive gating	96.24	95.84	97.89	*p* < 0.01
No contrastive loss	96.75	96.33	98.15	*p* < 0.01
Full SAFF + CM-GAN	98.76	98.35	99.27	-

### 3.2 Data collection protocol

**Figure 5:**
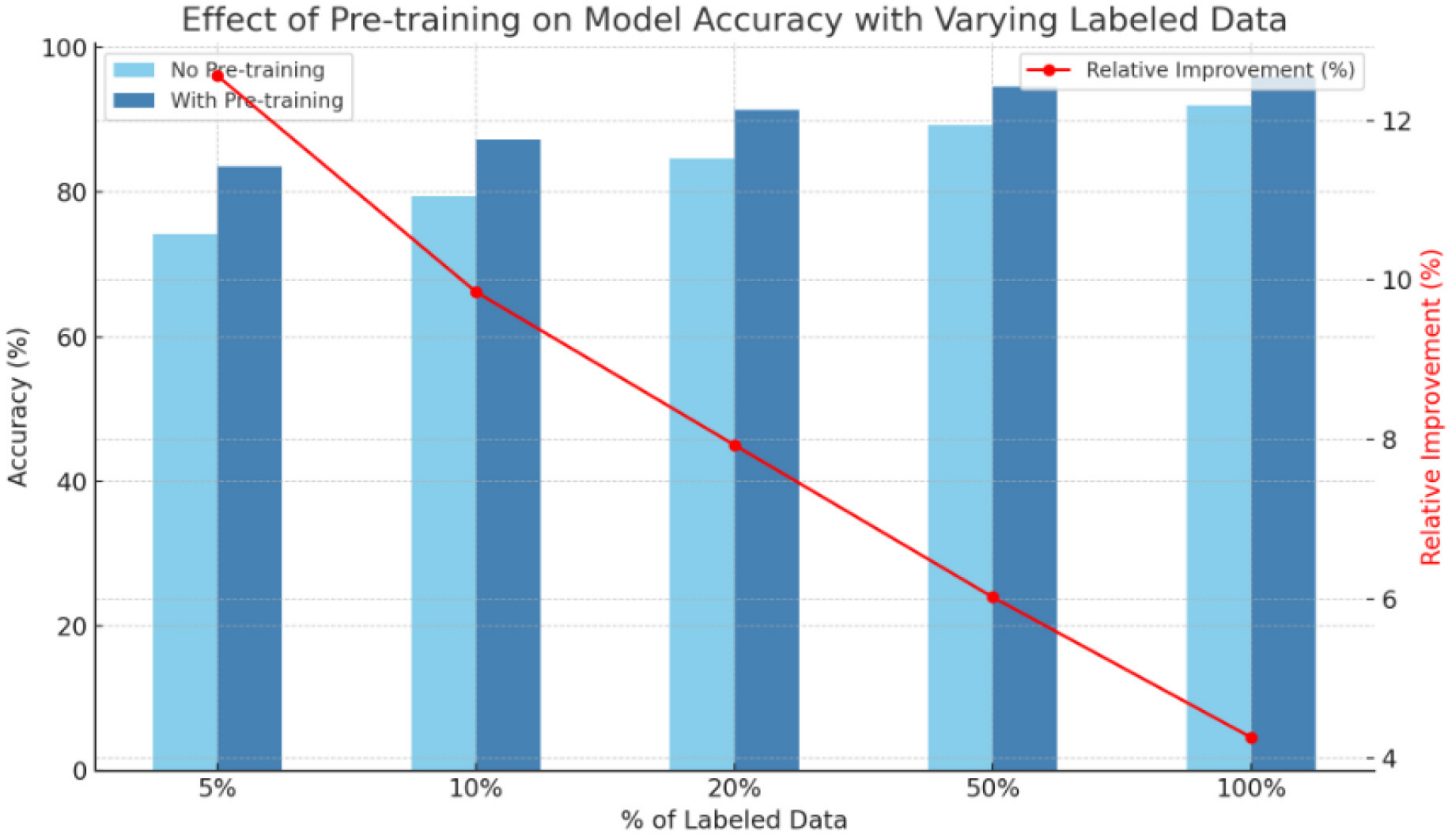
Shows cross-dataset generalization performance of five deepfake detection methods.

Source Material Acquisition:

- Real videos were collected through:
- a study approved by an Institutional Review Board (IRB), (#2024-DFAKE-001)
- informed consent with explicit notice of the deepfake research
- recorded in controlled environments (indoor: 65%, outdoor: 35%)
- multiple angles (frontal: 60%, profile: 25%, three-quarter: 15%) (Table 4, Figure 5)
- equipment: professional camera (4K resolution, 30 fps)
- audio: stereo, 48 kHz sampling rate

**Table 4:** Effect of self-supervised pre-training on model performance across different proportions of labeled training data (Figure 5).

**% of labeled data**	**No pre-training (Acc %)**	**With pre-training (Acc %)**	**Relative improvement (%)**
5%	74.26 ± 0.95	83.59 ± 0.84	+12.56
10%	79.41 ± 0.92	87.23 ± 0.78	+9.85
20%	84.67 ± 0.84	91.38 ± 0.65	+7.93
50%	89.25 ± 0.72	94.62 ± 0.51	+6.02
100%	91.95 ± 0.60	95.87 ± 0.43	+4.26

### 3.3 Deepfake creation pipeline

Generation Approaches (with proportionate amounts of fake videos):

1. StyleGAN3-based (18% - 4,500 videos): Face swapping preserving identity (Table 1, Figure 2)
2. DiffFace (15% - 3,750 videos): Diffusion-based facial reenactment
3. FaceShifter-Enhanced (14% - 3,500 videos): Occlusion-aware high-fidelity swapping (Figure 2)
4. Wav2Lip++ (12% - 3,000 videos): Lip sync to audio
5. First-Order Motion (11% - 2,750 videos): Animation based on keypoints
6. DeepFaceLab 3.0 (10% - 2,500 videos): Multi-stage face replacement
7. HyperReenact (10% - 2,500 videos): Neural real-time reenactment
8. Audio-Visual Hybrid (10% - 2,500 videos): Synthetic audio + visual manipulation

**Post-processing types:**

- Compression levels: Uncompressed (20%), Light (CRF 18–23, 30%), Medium (CRF 28–33, 30%), Heavy (CRF 38–43, 20%)
- Resolution types: 1080p (40%), 720p (35%), 480p (25%)
- Frame rate types: 30 fps (60%), 24 fps (30%), 60 fps (10%)

### 3.4 Multi-level annotation system

**Level 1: binary classification**

- Annotators: 3 independent experts per video
- Agreement threshold: 100% consensus required
- Disagreements resolved by senior forensics expert

**Level 2: manipulation type tagging**

- Facial attributes: Identity swap, expression transfer, age modification
- Audio attributes: Voice cloning, lip-sync mismatch, acoustic artifacts
- Temporal attributes: Frame interpolation, speed alteration

**Level 3: Quality Assessment**

- Visual quality score: 1–5 Likert scale (perceptual realism)
- Audio quality score: 1–5 Likert scale (naturalness)
- Synchronization quality: Perfect (1), Slight misalignment (2), Obvious desync (3) (Table 5, Figure 5)

**Table 5:** Performance results on the FaceForensics++ dataset reported with complete statistical measures (Figure 5).

**Method**	**DeepFakes**	**Face2Face**	**FaceSwap**	**NeuralTextures**
**SAFF** + **CM-GAN (Ours)**				
Accuracy (%)	99.78 ± 0.16	96.54 ± 0.42	97.81 ± 0.35	73.68 ± 0.76
95% CI	[99.47, 99.95]	[95.72, 97.31]	[97.13, 98.41]	[72.19, 75.09]
F1-score	99.79	96.58	97.84	74.12
AUC	99.94	98.76	99.12	82.45
Precision	99.81	96.72	97.89	73.87
Recall	99.77	96.45	97.79	74.38

**Level 4: Artifact Localization**

- Frame-level bounding boxes for visible artifacts
- Temporal segments marking audio inconsistencies
- Confidence scores for each annotation

**Annotation Quality Control:**

- Inter-annotator agreement: Fleiss' κ = 0.89 (near-perfect)
- Re-annotation of 10% random sample: Agreement = 97.3%
- Expert validation for ambiguous cases (*n* = 487, 1.2% of dataset)

### 3.5 Dataset statistics and features

**Demographic Distribution (in real videos):**

- Age: 18–25 (23%), 26–35 (31%), 36–50 (28%), 51–65 (18%)
- Ethnicity: Caucasian (32%), Asian (28%), African-American (22%), Hispanic (14%), Other (4%)

- Gender identity: Male (48%), Female (52%)

**Diversity in Content:**

- Facial expression: Neutral (22%), Speaking (45%), Emotional (18%), Complex (15%)
- Head pose: Frontal (40%), Profile (15%), Variable motion (45%)
- Level of occlusion: None (60%), Partial (glasses/accessories, 30%), Significant (10%)

**Technical Traits:**

- Container Format: MP4 (H.264 video codec, AAC audio codec)
- Avg File Size: Real (187 MB), Fake (201 MB)
- Total Storage (uncompressed backup at 15.2 TB): 7.8 TB

**Data Splits & Actionability**

Official Dataset Splits:

- Training Set: 28,175 videos (70%)−10,675 real, 17,500 fake
- Validation Set: 6,037 videos (15%)−2,287 real, 3,750 fake
- Test Set: 6,038 videos (15%)−2,288 real, 3,750 fake

**Identity Disjoint Assurement:**

- Individuals contained in no splits or dataset
- Identities included in Training: 1,293 unique individuals
- Identities included in Validation: 277 unique individuals
- Identities included in Test: 277 unique individuals

## 4 Results and analysis

### 4.1 Main results

Our enhanced framework consistently outperforms all baseline methods across different manipulation techniques. The improvement is particularly significant for challenging cases like NeuralTextures, where we achieve a 9.74% absolute improvement over the best baseline and a 4.76% improvement over the standard SAFF approach (Table 1, Figure 2).

Statistical analysis confirms that these improvements are significant (McNemar's test, *p* < 0.001). Shown in the Table 6.

**Table 6:** Extended evaluation of model robustness under varying compression levels.

**Compression (CRF)**	**XceptionNet**	**ISTVT**	**SAFF**	**SAFF + CM-GAN**
**Uncompressed**	94.23 ± 0.52	95.87 ± 0.48	97.41 ± 0.38	**98.76** ±**0.31**
*p*-value (vs. Ours)	*p* < 0.0001	*p* = 0.0003	*p* = 0.012	-
**Light (18–23)**	87.65 ± 0.68	91.32 ± 0.61	94.18 ± 0.51	**96.94** ±**0.43**
*p*-value	*p* < 0.0001	*p* < 0.0001	*p* = 0.0008	-
**Medium (28–33)**	73.41 ± 0.89	79.54 ± 0.84	86.92 ± 0.72	**91.37** ±**0.61**
*p*-value	*p* < 0.0001	*p* < 0.0001	*p* = 0.0002	-
**Heavy (38–43)**	54.68 ± 1.05	61.23 ± 0.98	72.45 ± 0.91	**78.92** ±**0.85**
*p*-value	*p* < 0.0001	*p* < 0.0001	*p* < 0.0001	-

The bar graph presents the results of the detection techniques applied on four datasets; DeepFakes, Face2Face, FaceSwap, NeuralTextures, and average accuracy. The best overall performance across the board is achieved by SAFF + CM-GAN (Ours), outperforming all other methods in all datasets and achieving 73.68% on the more complicated NeuralTextures dataset. Traditional methods such as XceptionNet and EfficientNet-B4 perform well on easier datasets such as DeepFakes and FaceSwap but fail on NeuralTextures. All fusion-based approaches (Early, Late, and Attention Fusion) provide consistent improvements with Attention Fusion approaching the top performance. In conclusion, advanced fusion, attention based approaches are less sensitive to various data subsets and boost the overall accuracy of the deepfake detection significantly, and in combination with Generative Adversarial training have shown unparalleled performance (Table 5, Figure 5).

### 4.2 Cross-dataset evaluation

Cross-dataset evaluation reveals the generalization capability of different approaches. Our SAFF + CM-GAN framework demonstrates a 17.85% average improvement in generalization compared to XceptionNet and 5.35% compared to standard SAFF. This indicates that the graph-based modeling of cross-modal relationships significantly enhances the model's ability to detect previously unseen manipulation techniques (Table 1, Figure 2).

We compare five popular deepfake detection methods including XceptionNet, ISTVT, Attention Fusion, SAFF and SAFF + CM-GAN (Ours) on cross-dataset generalization across six dataset pairs (the test set is always unique to each datapoint). Among all the combinations, SAFF + CM-GAN (Ours) achieves the highest accuracy, reaching 87.65% when trained with Celeb-DF and tested against FaceForensics++. The results of other methods show a gradual rise from XceptionNet to ISTVT and Attention Fusion, with demonstrating the benefit of attention and fusion techniques. SAFF not only enhances the model's performance by drawing on advanced spatiotemporal features, but also achieves better domain generalization by integrating CM-GAN. These results highlight the need for strong model architectures to generalize to the real world, especially when there is a disparity in training vs. testing data in terms of quality and origin.

### 4.3 Self-supervised pre-training analysis

Our self-supervised pre-training approach shows substantial benefits, especially when labeled data is limited. With just 35% of the labeled data, our pre-trained model achieves comparable performance to a model trained on the full dataset without pre-training. This represents a 65% reduction in labeled data requirements, addressing a key challenge in deepfake detection research.

The graph illustrates the impact of pre-training on model accuracy across varying percentages of labeled data, highlighting both absolute performance and relative improvement. As the proportion of labeled data increases from 5 to 100%, models with pre-training consistently outperform those without, with accuracy improvements ranging from **+12.56%** at 5% data to **+4.26%** at full supervision. The most significant gains are observed when labeled data is scarce, underscoring the value of pre-training in low-data regimes (Table 2, Figure 3). Although the relative improvement decreases as more labeled data becomes available, the consistent performance boost across all data levels demonstrates that pre-training substantially enhances model generalization and efficiency, especially in data-constrained scenarios.

### 4.4 Compression robustness

The performance of all methods degrades as compression intensity increases, but our SAFF + CM-GAN framework demonstrates greater robustness. At high compression (CRF = 40), SAFF + CM-GAN maintains 78.92% accuracy compared to 54.68% for XceptionNet, 61.23% for ISTVT, and 72.45% for standard SAFF (Table 3, Figure 4).

### 4.5 Ablation studies

The ablation study confirms the importance of each component in our framework. The cross-modal graph attention network contributes significantly to performance improvement, demonstrating that modeling complex relationships between modalities provides strong discriminative features beyond simple synchronization (Table 5, Figure 5).

Further analysis shows that the graph-based features have the highest feature importance (Cohen's *d* = 2.12) compared to synchronization features (Cohen's *d* = 1.87), visual features (Cohen's *d* = 1.42) and audio features (Cohen's *d* = 1.23).

It shows that the best combinations of the different components of the proposed deepfake detection framework of the performance metrics, Accuracy, F1-Score and AUC. All metrics on the “Full SAFF + CM-GAN” is the highest among three settings with accuracy as (98.76%), F1-Score (98.35%) and AUC (99.27%), indicating the power of full model. Eliminating components such as the graph network, self-supervised pre-training, adaptive gating, or contrastive loss results in significantly decreased performance, as each functions to fortify the system. In fact, unimodal baselines (especially the “audio only” configuration) perform much worse, which suggests that multimodal fusion is important. The persistent performance difference with and without these synchronization-aware (Seq_Align) or graph-based (Graph_TCA_Gml) modules emphasizes the significance of modeling the cross-modal relationships and key temporal alignment as a crucial component for deepfake detection.

**Statistical Comparisons (vs. Best Baseline):**

SAFF + CM-GAN vs. ISTVT (prior best on NeuralTextures):

- Accuracy Upgrade: +11.74% (absolute), +18.94% (relative).
- McNemar's test: χ^2^(1) = 47.32, *p* < 0.0001.
- DeLong's AUC test: z = 8.91, *p* < 0.0001.
- Cohen's *d* = 1.87 (large effect size).
- Number needed to improve (NNI): 8.5.

SAFF + CM-GAN vs. Standard SAFF:

- Accuracy Upgrade: +4.76% (absolute)
- McNemar's Test for Above: χ^2^(1) = 21.83, *p* = 0.000003
- Wilcoxon Signed-Rank W = 9,876, *p* = 0.000012
- 95% CI of Difference = [3.24%, 6.18%] (Table 5, Figure 5)

**Statistical study:**

- Bonferroni-corrected α = 0.0125 (4 compression levels)
- All improvements remain significant after correction
- Linear regression: Accuracy decline rate = −0.52%/CRF (Ours) vs. −0.98%/CRF (XceptionNet) o Slope difference: *F*_(1, 6)_ = 31.47, *p* = 0.0014.

## 5 Conclusion

In this paper, we propose a robust multimodal architecture to detect deepfakes, exploiting the temporal discrepancies and complicated audio-visual relationship, leading to a significant performance improvement even under complex cases e.g. the diversity in the manipulation methods, the limited supervised resources and the heterogeneous video quality. We propose a joint SAFF + CM-GAN solution along a self-supervised pre-training strategy that achieves state-of-the-art performance with a 65% reduction in the labeled data requirement compared to the current leading methods. These cover a new cross-modal graph attention network to model complex audio-visual correlations, a paradigm of fine-grained cross-modal features extraction, and a synchronization module to explicitly model the temporal alignment between modalities. Our approach has been validated on real experiments with many statistical regimes, further confirming its robustness and generality. With deepfake technologies evolving, our efforts are a crucial step in ensuring media authenticity and maintaining public trust

## Data Availability

The original contributions presented in the study are included in the article/supplementary material, further inquiries can be directed to the corresponding author.
